# Bibliometric analysis of global research trends and development prospects of *Smilax glabra* Roxb using CiteSpace

**DOI:** 10.3389/fpls.2025.1651650

**Published:** 2025-09-30

**Authors:** Wenqing Shi, Xiao Li, Xin Zhao, Jingjing Jiang, Chenxi Wang, Guorong Fan, Yuefen Lou

**Affiliations:** ^1^ Department of Pharmacy, Shanghai Fourth People’s Hospital, School of Medicine, Tongji University, Shanghai, China; ^2^ School of Medicine, Shanghai University, Shanghai, China; ^3^ Department of Clinical Pharmacy, Shanghai General Hospital, Shanghai Jiaotong University School of Medicine, Shanghai, China; ^4^ Department of Clinical Pharmacy, Shanghai Jiao Tong University School of Medicine, Shanghai, China; ^5^ School of Pharmacy, Shanghai Jiao Tong University, Shanghai, China

**Keywords:** *Smilax glabra* Roxb, Citespace, visual analysis, knowledge maps, research hotspots

## Abstract

**Introduction:**

*Smilax glabra* Roxb (SGR) is extensively utilized in the management of disorders, including hyperuricemia and gout, due to its notable pharmacological effects, and it is also the primary component in the functional food turtle jelly. Despite extensive research on SGR, no systematic statistical analysis of the literature has been conducted on it. This study provides a comprehensive bibliometric analysis of SGR, identifies the current research landscape, identifies hotspots, and performs trend analysis.

**Methods:**

All Chinese and English literatures pertaining to SGR was gathered from Web of Science Core Collection (WoSCC), China Knowledge Network (CNKI), Wanfang database, and VIP database, subsequently de-duplicated and organized, with CiteSpace software employed for visualization and analysis of the literature.

**Results:**

A total of 1723 articles were incorporated into the analysis, and the quantity of SGR-related publications in English persists in its upward trajectory. Sun Weifeng, Lisa Dong, and Zhang Qingfeng emerged as the principal contributors, while Beijing University of TCM and Zhejiang University of TCM established themselves as the foremost publishing organizations. Noteworthy, keywords indicative of contemporary research focal points encompassed “Chinese medicine treatment,” “gout,” “anti-inflammatory,” and “network pharmacology.”

**Discussion:**

The investigation into SGR concentrates on pharmaceuticals and their active components, therapeutic interventions, and pharmacological mechanisms. Recently, anti-inflammatory and cyberpharmacology have emerged as prominent trends, indicating that the integration of animal studies with molecular bioinformatics to investigate the pharmacological mechanism of SGR is the main research direction in the future, while cardiovascular protection and neuroprotective effects have become significant areas of recent inquiry. Consequently, SGR is anticipated to be a functional plant for the treatment of various diseases.

## 1 Introduction

*Smilax glabra* Roxb (SGR) is classified within the genus Smilax of the Liliaceae family, and is recognized for clearing heat and removing dampness, detoxifying, and decongesting properties (Haohan et al., 2023; Shuang et al., 2021). SGR is a prevalent component in TCM, acknowledged for its dual function as both a food and a medicinal material, and possesses a broad spectrum of therapeutic properties. The chemical composition of SGR is intricate, predominantly comprising of sterols, flavonoids, flavonoid glycosides, phenols, sugars, and additional constituents (Shi et al., 2023). SGR is commonly employed in TCM to address ailments including damp heat sores, eczema, arthralgia, and syphilis (Mengqi and Deping, 2020). Contemporary pharmacological research has established that SGR exhibits multiple pharmacological properties, including anti-tumor (Guo et al., 2022), antioxidant (Nguyen et al., 2024), anti-inflammatory (Shi et al., 2020), detoxification effect (Zhao et al., 2025), and uric acid-lowering effects (Huang et al., 2019). Moreover, SGR serves as the primary component in numerous functional foods, particularly tortoise jelly and Tu Fu Ling tea. Furthermore, SGR health soup is effective in dispelling dampness, detoxifying, and strengthening the spleen, demonstrating its significant application value in various diseases and health foods.

Astilbin, a dihydroflavonoid prevalent in numerous plants, is the predominant active compound in the SGR, and the Chinese Pharmacopoeia uses the astilbin content to assess the quality of these herbs, stipulating a minimum concentration of 0.45% in the SGR. Contemporary pharmacological research has demonstrated that lokusinoside possesses multiple potential biological activities. Furthermore, astilbin demonstrates anticancer (Sun et al., 2018), anti-inflammatory (Chen et al., 2020), hepatoprotective (Yang et al., 2024), antidepressant (Yao et al., 2022) and anti-aging effects (Zhang et al., 2021). Consequently, astilbin possesses the potential to serve as a nutritional supplement, reflecting the transformation of TCM into contemporary pharmaceuticals, and has a wide range of developmental values and application prospects. Notwithstanding the advantageous pharmacological properties of loksinoside, its chemical instability and poor oral bioavailability restrict the extensive clinical application of astilbin. As the main plant source of astilbin, SGR has important applications as both a functional food and therapeutic drug, but the current status of SGR research has not been systematically bibliometrically tallied and summarized.

Bibliometrics involves the utilization of mathematical and technical methods to quantitatively analyze literature and measure the influence of academic accomplishments and scientific advancements.Its primary goal is to deliver objective and reliable research outcomes via quantitative analysis, aiding scholars and policymakers in comprehending the current status and developmental trajectory of this academic field (Min et al., 2022). Bibliometrics uses statistical techniques to analyze data from different types of published research papers, including books, journal articles, and datasets, and presents the results of the analyses in the form of tables and graphical visualizations to elucidate the correlations between different academic publications, while high-impact research results can be pinpointed and understood directly through these statistics (Manoj Kumar et al., 2022). In addition, bibliometrics can reveal current developments and challenges in subject areas, thus providing a global view and analysis of current hot research and core challenges (Lubowitz et al., 2023). CiteSpace is an information visualization analysis tool primarily used for citation analysis of scientific literature and the development of knowledge graphs. With advanced data processing and visualization capabilities, it can comprehensively analyze the hotspots, trends, and dynamic shifts within the academic landscape (Donthu et al., 2021; Zhang et al., 2024). CiteSpace is widely used for bibliometric analysis of herbal medicines and natural products, as it provides an effective way to simplify and visualize complex research analysis by identifying landmark nodes, pivot points, and hub nodes (Lai et al., 2024). Our study employs CiteSpace as a tool to visually present and map the knowledge structure of the literature, aiming to systematically and comprehensively elucidate the latest research progress and future emerging trends in SGR.

This study presents the inaugural bibliometric analysis of SGR-related literature utilizing the WoSCC, CNKI, Wanfang, and VIP databases from 2000 to 2024, investigating research hotspots and developmental trajectories over the past 24 years. The visualization and analysis process of SGR-related literature were conducted using the CiteSpace platform, encompassing a thorough evaluation of the dimensions of publication frequency, research authors, collaborating institutions, keywords, and highly cited works. This paper’s primary contributions encompass (1) forecasting future publication trends by summarizing the publication frequency of SGR-related publications; (2) examining the co-occurrence relationships and geographic distribution of major research domains while predicting viable collaboration; and (3) pinpointing prominent research trends in the field through keyword research and citation analysis, which will provide a foundation for future clinical research and rational drug utilization.

## 2 Materials and methods

### 2.1 Data source

**Figure 1:**
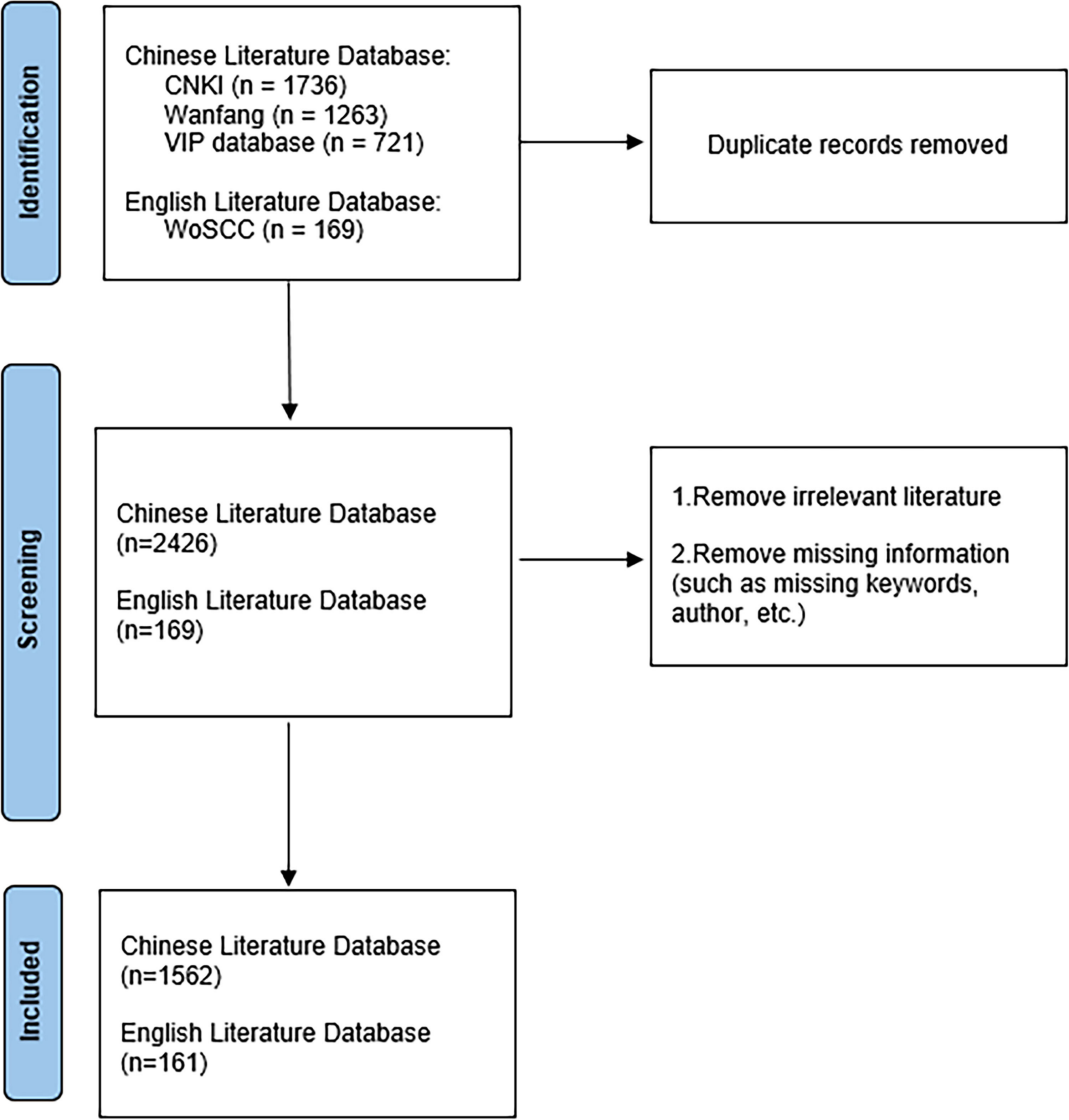
Flowchart of CiteSpace bibliometric analysis.

The WoSCC database, encompassing extensive information on more than 10,000 journals from all over the world, was utilized to investigate English literature, serving as the predominant platform for scientific research (Wang, 2024). Access to Chinese literature includes CNKI, Wanfang, and the VIP database, which are currently the largest information platforms for Chinese journals at present. The WoSCC search term was “Topic= (Smilax glabra Roxb or Smilax glabra Roxb. or tufuling or Smilacis Glabrae Rhizoma)”, set the publication time as January 1, 2000, to December 31, 2024, set the language as “English”, and limited the types of articles to “article” and “review article”. According to the title and keywords facilitated the refinement and exportation in the format of a plain text file, and the search and analysis work flow is illustrated in **Figure 1**.

### 2.2 Data sorting

The literature obtained from Chinese databases was imported into NoteExpress software for duplicate elimination and manually screened according to the classification criteria. The included literature was exported in RefWorks format for further analysis, whereas the English literature was directly exported in the full-text format. The inclusion criteria were as follows: (1) the literature encompassed drug trials, animal experiments, and clinical investigations pertaining to SGR; and (2) comprised both research papers and review articles. Finally, it was ultimately verified that there were no duplicate documents within the 1723 documents.

### 2.3 Data analysis and results visualization

GraphPad 8.0 was employed to analyze the annual publication frequency of the literature, and the bibliometric software CiteSpace 6.3.1 was used to visualize and analyze all the collected literature. In the CiteSpace analysis, we analyzed with a 1-year time slice. The node types were selected as co-authors, institutions, and keywords, respectively. “N citations with the highest number of citations in the slice (TopN)” and “Top N% citations with the highest number of citations in the slice (TopN%)” are default values. “Threshold (g-index)” is set to 25. When analyzing Chinese literature, set the clipping method and check the options of “pathfinder” and “pruning sliced networks,” while when analyzing English literature, it is not required. Collaborative network analysis facilitated the depiction of collaborative relationships between authors, institutions, and countries, enabling the investigation of geographic distribution and network structure related to the SGR research domain. The keywords in the literature were also analyzed for co-occurrence and clustering to explore the relationships between keywords. This analysis produces a keyword co-occurrence map that elucidates the fundamental themes of the research field and their interrelations. Each node in the visualization graph represents an author, an institution, and a keyword; the size of the node indicates the number of papers published; the larger the node, the higher the number of papers issued; and the connecting lines between the nodes indicate the connection between different authors, institutions, or keywords. The closer the node color is to red, the shorter the publication time.

## 3 Results

### 3.1 Literature search results

A search formula was employed for article acquisition, and 3124 documents were retrieved in this study, including 2951 in Chinese and 173 in English. A total of 1736 articles were obtained from the CNKI database, 1263 results were retrieved from the Wanfang database, 721 results were retrieved from the VIP database, and 169 results were retrieved from the WoSCC database. Based on the exclusion criteria, a total of 1401 duplicate articles were eliminated, resulting in the inclusion of 1562 articles (90.7%) in Chinese and 161 articles (9.3%) in English, culminating in a total of 1723 articles.

### 3.2 Literature volume and trend analysis

**Figure 2:**
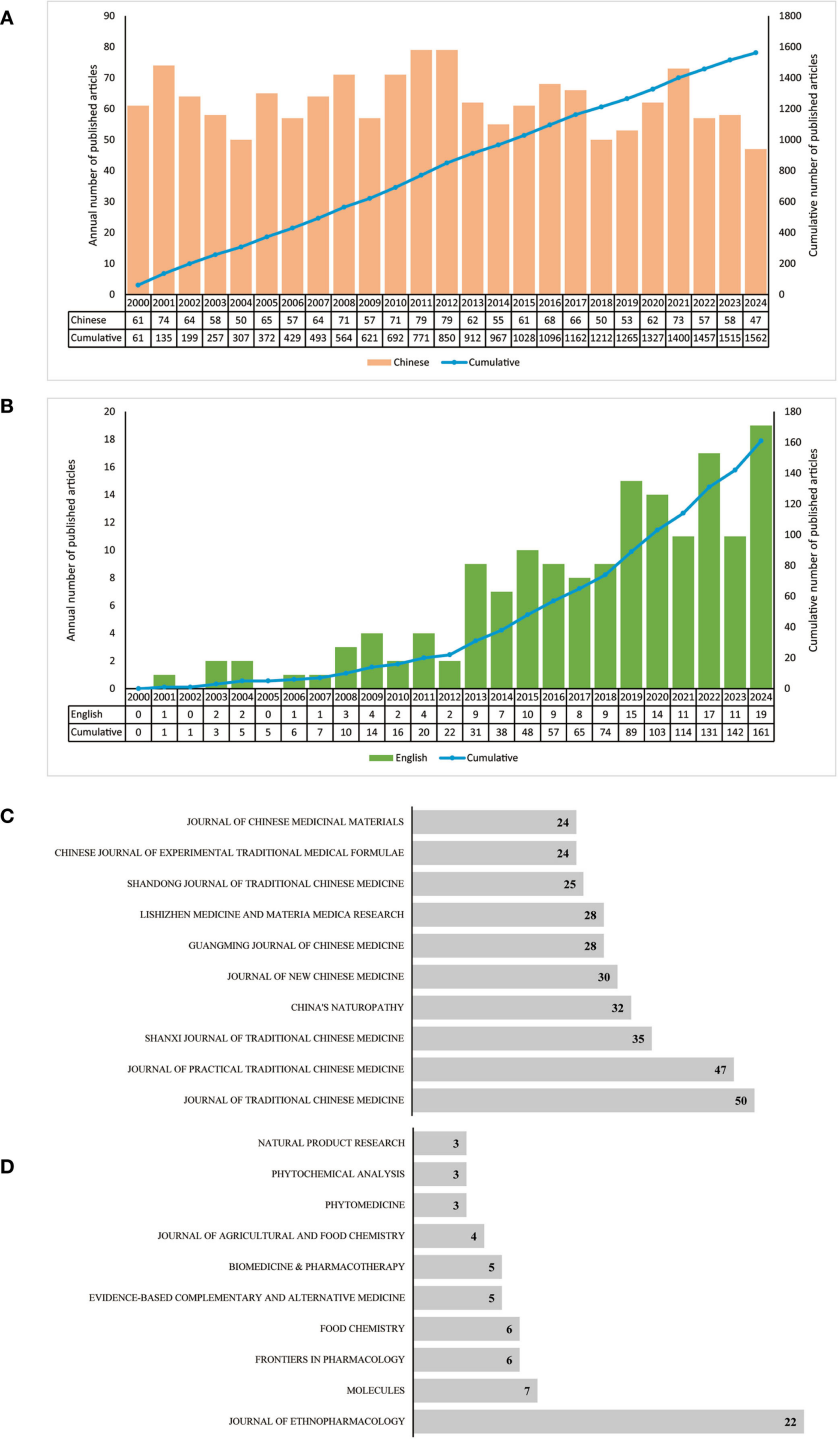
Annual trend of publications of SGR: the yearly publication volume of Chinese **(A)**, and English literature **(B)**; the top 10 journals for Chinese **(C)**, and English publications **(D)**.

The yearly publication volume and trend of the 1723 papers analyzed in this study are illustrated in **Figures 2A**, **2B**. **Figure 2A** illustrates a distinct upward trajectory in the cumulative publication volume of Chinese articles. Conversely, the cumulative publication of Chinese articles increased rapidly from 61 in 2000 to 1562 in 2024, clearly illustrating the significance and dynamism of SGR in TCM research. **Figure 2B** illustrates the cumulative publication data of English literature, indicating an upward trend in cumulative publications 2000 to 2024, but the growth rate is relatively slow. Beginning 2013, the publication of SGR articles in English experienced a substantial rise, culminating in a peak of annual publication in 2019, and, from 0 articles in 2000 to 161 articles in 2024, indicating that SGR is garnering attention from multiple countries, and its international influence is expanding. **Figures 2C**, **2D** illustrate the top ten leading journals in terms of annual publication volume in Chinese and English, respectively. The Journal of TCM (N = 50) and Journal of Ethnopharmacology (N = 22) are the journals with the highest number of publications in Chinese and English, respectively, which demonstrated the wide recognition of SGR in the field of TCM research. Generally, these results highlight the growing international connections and academic attention of SGR.

### 3.3 Co-author analysis

**Figure 3:**
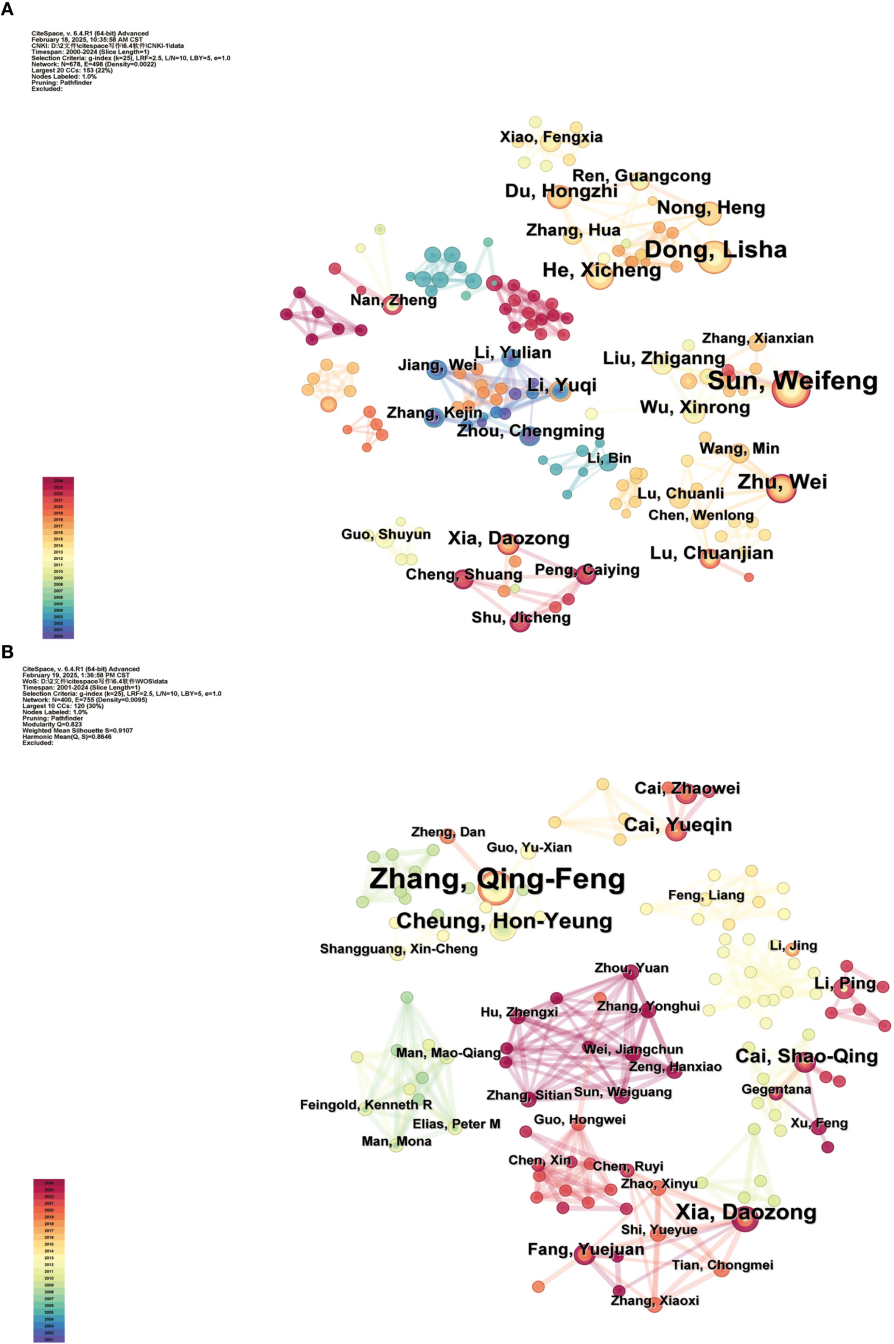
Cooperative network of authors in Chinese **(A)** and English **(B)** literature on SGR.

This study analyzed the authors of the literature Chinese literature produced 678 nodes and 498 connections, yielding a network density of 0.0022. Conversely, the English literature generated 400 nodes and 755 connections, resulting in a network density of 0.0095. **Figures 3A**, **3B** depict the co-occurrence maps of the authors in the Chinese and English literature, respectively. In these maps, nodes symbolize authors, with node size denoting the quantity of publications by the authors. Lines illustrate collaborative relationships between authors, and the thickness of the lines reflects the degree of cooperation among them (Chen, 2006; Mokhnacheva and Tsvetkova, 2020). **Table 1** presents the ten foremost authors. The authors with the most publications in Chinese literature are Sun, Weifeng (21 papers), Dong, Lisha (17 papers), and Zhu, Wei (11 papers). The prominent authors in English literature comprise Zhang, QingFeng (10 articles), Xia, Daozong (5 articles), and Cheung, HonYeung (5 articles), among others.

**Table 1:** Top 10 authors of Chinese and English literature.

Chinese literature		English literature	
Author	Number of published papers	Author	Number of published papers
Sun, Weifeng	21	Zhang, QingFeng	10
Dong, Lisha	17	Xia, Daozong	5
Zhu, Wei	11	Cheung, HonYeung	5
He, Xicheng	10	Cai, ShaoQing	4
Xia, Daozong	8	Li, Li	4
Nong, Heng	8	Cai, Yueqin	4
Li, Yuqi	8	Cai, Zhaowei	3
Du, Hongzhi	8	Li, Ping	3
Wu, Xinrong	7	Fan, Guorong	3
Lu, Chuanjian	7	Li, Bo	3

### 3.4 Contribution of institutes

**Figure 4:**
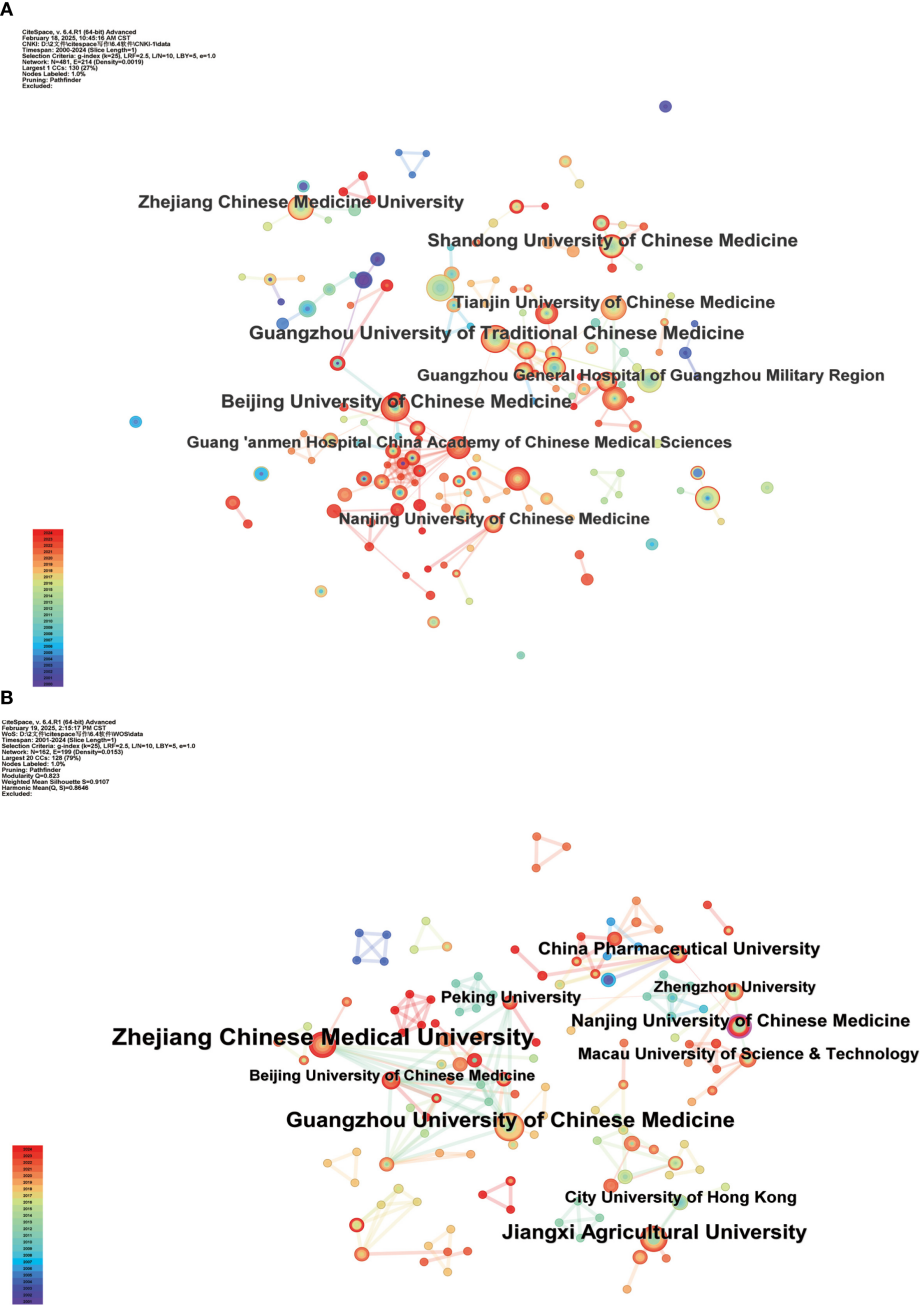
Co-occurrence map of Chinese **(A)** and English **(B)** literature on SGR cooperative institutions.

This study involved the analysis of Chinese and English literature pertaining to Traditional TCM. SGR was analyzed by collaborating institutions, resulting in the creation of a co-occurrence atlas of these institutions. **Figures 4A**, **4B** depict the co-occurrence atlas for Chinese and English literature institutions, respectively. **Table 2** enumerates the ten leading institutions according to their publication count. The atlas of institutions linked to Chinese literature comprised 481 nodes and 214 connections, yielding a density of 0.0019. Beijing University of Chinese Medicine had the highest publication count, totaling 26 articles. The English literature atlas comprised 162 nodes and 199 links, resulting in a density of 0.0153. Zhejiang Chinese Medical University had the highest number of publications in this category, totaling 16.

**Table 2:** The top 10 institutions with the number of Chinese and English literature publications.

Chinese literature		English literature	
Institutions of publications	Number of published papers	Institutions of publications	Number of published papers
Beijing University of Chinese Medicine	26	Zhejiang Chinese Medical University	16
Guangzhou University of Traditional Chinese Medicine	25	Guangzhou University of Chinese Medicine	12
Shandong University of Chinese Medicine	23	Jiangxi Agricultural University	11
Zhejiang Chinese Medicine University	22	Nanjing University of Chinese Medicine	7
Guiyang College of Traditional Chinese Medicine	19	China Pharmaceutical University	7
Tianjin University of Chinese Medicine	19	Macau University of Science & Technology	6
Guang ‘anmen Hospital China Academy of Chinese Medical Sciences	16	City University of Hong Kong	6
Guangzhou General Hospital of Guangzhou Military Region	16	Peking University	6
Nanjing University of Chinese Medicine	16	Beijing University of Chinese Medicine	5
Hubei University of Chinese Medicine	14	Zhengzhou University	5

### 3.5 Research hotspot analysis

#### 3.5.1 The most cited publications

**Figure 5:**
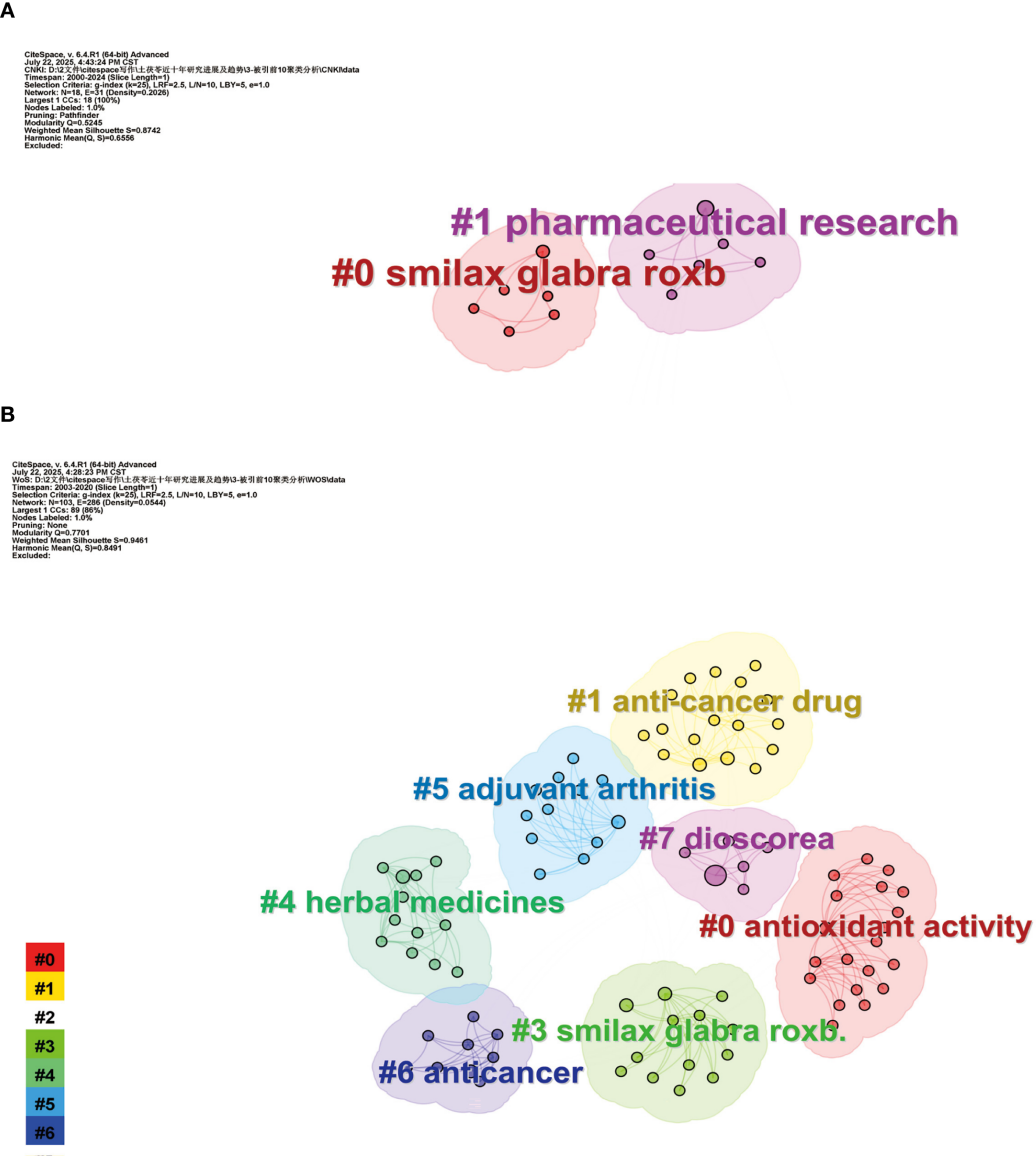
Publications clustering analysis of the top 10 keyword-based literature in Chinese **(A)** and English **(B)**.

The analysis of the top 10 cited articles allows us to quickly identify emerging trends and potential research directions in the academic field. This study identified the top 10 most cited papers both Chinese and English, which have significantly contributed to the research history of SGR (**Table 3**). The most frequently cited paper was the review by Wang Jianping et al. published in 2013, which received 391 citations. The article meticulously delineated the chemical makeup and pharmacological effects of SGR, establishing a crucial foundation for later investigations into the pharmacological mechanisms of SGR. The most often referenced work in English literature is the study authored by Itharat, Arunporn, et al. This study detailed the anticancer activities of various plants, including SGR, on cancer cells and compared the cytotoxicity of different solvent extracts, offering theoretical support for clinicians in the selection and rationalization of cancer treatment medications. The clustering analysis of the top 10 keyword-based literature in Chinese and English is shown in **Figures 5A**, **5B**. Following the grouping and analysis of the literature based on keywords, it was categorized into eight thematic clusters, including Smilax glabra Roxb, pharmaceutical research, antioxidant activity, anti-cancer drug, herbal medicines, adjuvant arthritis, anticancer, and dioscorea. The above findings indicate that the prevailing research emphasis in the domain of SGR is mostly on antioxidant activity and anticancer effects.

**Table 3:** The Top 10 Cited References.

Chinese literature					English literature				
Code	Title	Author	Year	Number of citations	Code	Title	Author	Year	Number of citations
1	Research Progress on the Chemical Composition and Pharmacological Effects of Smilax glabra Roxb	Wang Jianping	2013	391	1	*In vitro* cytotoxic activity of Thai medicinal plants used traditionally to treat cancer	Itharat, Arunporn	2004	208
2	Study on the Anti-inflammatory, Analgesic and Diuretic Effects of Smilax glabra Roxb and Astilbin	Zhang Baijia	2004	247	2	Anti-tumor activities of active ingredients in Compound Kushen Injection	Wang, Wei	2015	160
3	Professor Zhu Liangchun’s experience in treating gouty arthritis is introduced	Tian Hua	2010	222	3	Structural characterization and macrophage immunomodulatory activity of a novel polysaccharide from Smilax glabra Roxb	Wang, Min	2017	137
4	Lu Zhizheng’s experience in treating gout arthralgia	Shi Ruifang	2011	211	4	Immunomodulatory activity of the aqueous extract from rhizome of Smilax glabra in the later phase of adjuvant-induced arthritis in rats	Jiang, Jieyun	2003	107
5	Modern research progress of the traditional Chinese medicine Smilax glabra Roxb	Cheng Shuang	2021	204	5	Novel applications of mass spectrometry-based metabolomics in herbal medicines and its active ingredients: Current evidence	Wang, Xijun	2019	83
6	The active components and pharmacological mechanisms of Smilax glabra Roxb are studied based on network pharmacology	Yong Chen	2019	163	6	Protective effect of Smilax glabra extract against lead-induced oxidative stress in rats	Xia, Daozong	2010	83
7	Screening study on the Inhibition of URAT1 expression and uric acid-lowering Effects of Traditional Chinese Medicines such as Smilax glabra Roxb	Sun Hong	2012	157	7	Antioxidant activity of Rhizoma Smilacis Glabrae extracts and its key constituent-astilbin	Zhang, Qing-Feng	2009	79
8	An Overview of the Pharmaceutical Research on Smilax glabra Roxb	Fan Jiumei	2018	151	8	Astilbin: a promising unexplored compound with multidimensional medicinal and health benefits	Sharma, Abhishek	2020	76
9	Experimental Study on the Anti-inflammatory and Analgesic Effects of Smilax glabra Roxb Injection	Sun Xiaolong	2004	145	9	Protective effects of Rhizoma smilacis glabrae extracts on potassium oxonate- and monosodium urate-induced hyperuricemia and gout in mice	Liang, Guoyan	2019	75
10	Professor Wang Yuxi’s experience in treating eczema	Yan Jingdong	2005	141	10	Taxifolin Suppresses UV-Induced Skin Carcinogenesis by Targeting EGFR and PI3K	Oi, Naomi	2012	74

#### 3.5.2 Keyword co-occurrence analysis

**Figure 6:**
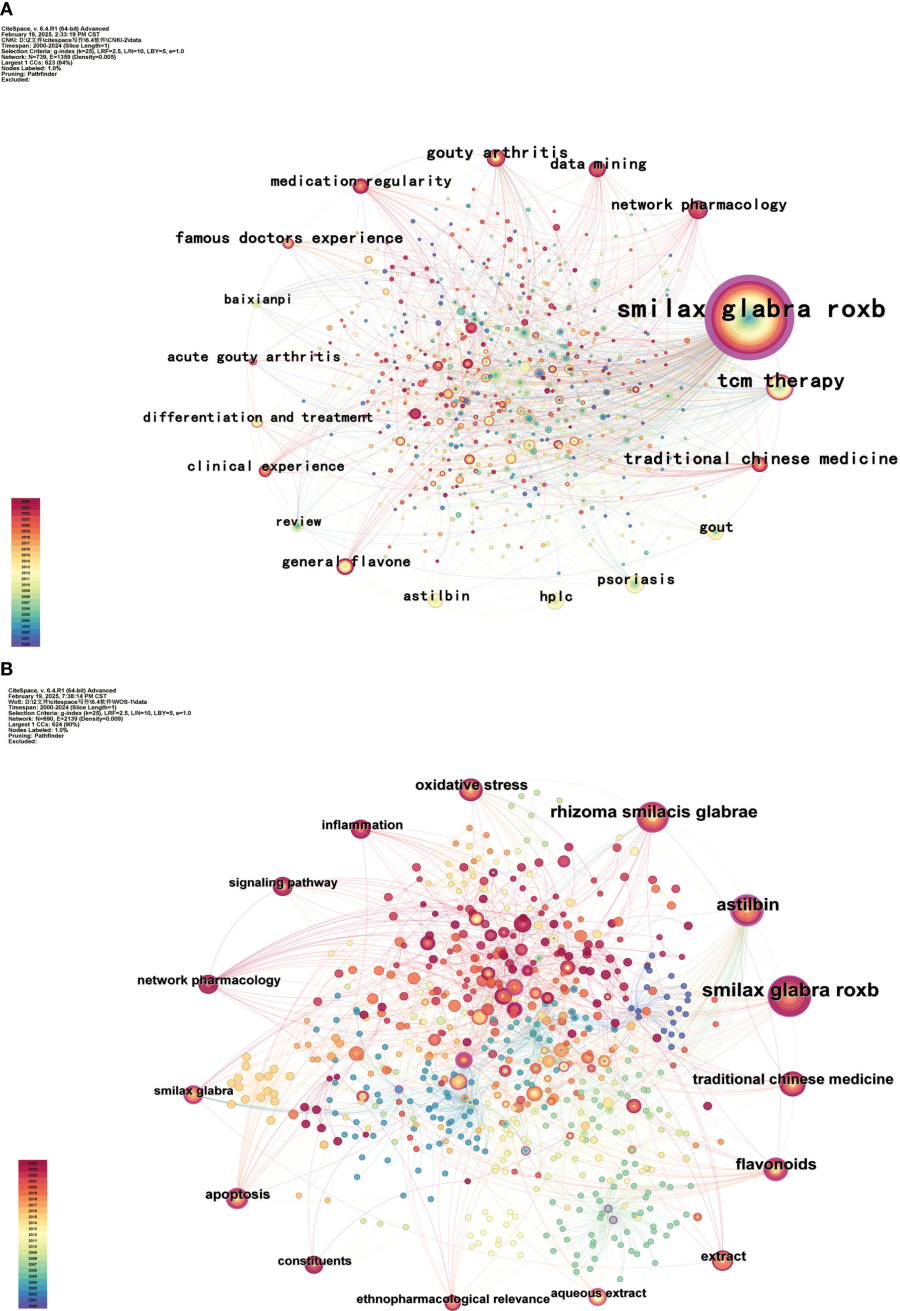
Co-occurrence of key words in Chinese **(A)** and English **(B)** related literature on SGR.

Analyzed the co-occurrence of keywords pertaining to TCM and SGR, yielding a keyword co-occurrence map with 739 nodes, 1,359 connections, and a density of 0.005. The keyword co-occurrence map of keywords in English literature, comprising 690 nodes, 2,139 connections, and a density of 0.009, is illustrated in **Figures 6A**, **6B**. **Table 3** illustrates that the predominant keywords in the Chinese literature are SGR (677 occurrences), TCM therapy (147 occurrences), gout (87 occurrences), astilbin (74 occurrences), and TCM (60 occurrences). Conversely, the high-frequency keywords in the English literature are SGR. (59 occurrences), astilbin (40 occurrences), rhizoma smilacis glabrae (38 occurrences), flavonoids (28 occurrences), and TCM (21 occurrences), as outlined in **Table 4**.

**Table 4:** High-frequency keywords in Chinese and English literature related to SGR.

Chinese literature			English literature		
Keyword	Frequency	Centrality	Keyword	Frequency	Centrality
smilax glabra roxb	677	0.85	smilax glabra roxb	59	0.1
tcm therapy	147	0.14	astilbin	40	0.4
gout	87	0.1	rhizoma smilacis glabrae	38	0.19
astilbin	74	0.16	flavonoids	28	0.13
traditional chinese medicine	60	0.1	traditional chinese medicine	21	0.05
psoriasis	59	0.11	oxidative stress	19	0.1
gouty arthritis	56	0.07	apoptosis	16	0.17
hyperuricemia	48	0.04	extract	16	0.06
HPLC	47	0.03	network pharmacology	14	0.05
famous doctors experience	45	0.04	inflammation	13	0.05

#### 3.5.3 Key words cluster analysis

**Figure 7:**
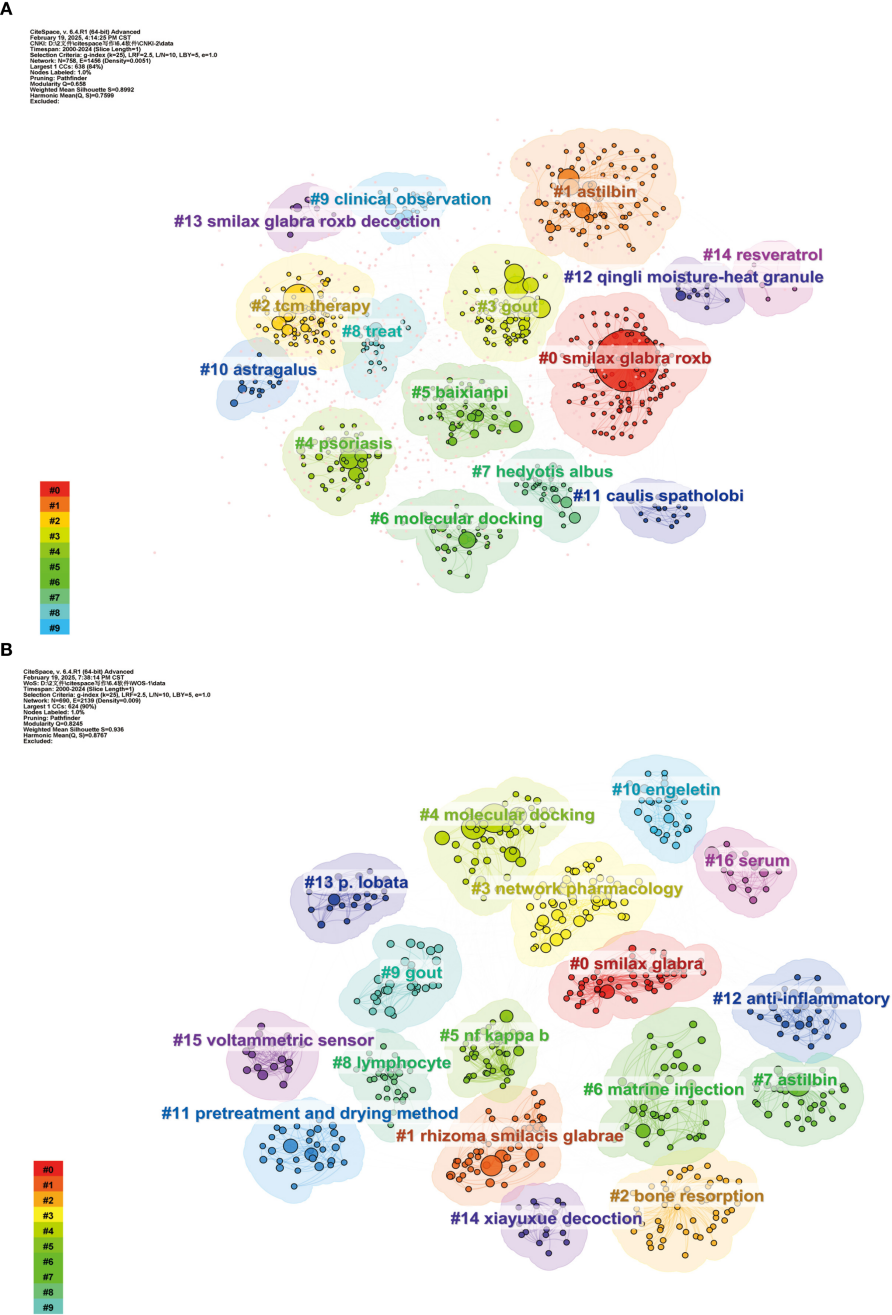
Keyword clustering of Chinese **(A)** and English **(B)** literature related to SGR.

Modularity (Q-value) signifies the importance of the cluster structure within the atlas. Generally, a Q value > 0.3 suggests that the cluster structure is substantial. The Silhouette (S value) assesses the validity of the clustering results; it is widely accepted that an S > 0.7 is generally regarded as indicative of credible clustering results (Chun et al., 2024). This study conducted a cluster analysis on keywords associated with SGR, with results illustrated in **Figures 7A**, **7B**. The Q values for keyword clustering in the Chinese and English literature were 0.658 and 0.8992, respectively. The S values were 0.8245 and 0.936, respectively, signifying that the keyword clustering results of this study were highly reliable. There are 15 clustering labels in the Chinese literature atlas. Research concentrates on pharmacological agents and their chemical constituents (#0 SGR., #1 astibin, #5 baixianpi, #12 qingli moisture-heat granule, #13 SGR.), #14 resveratrol, etc.); disease research (#3 gout, #4 psoriasis); and mechanism research (#6 molecular docking). Seventeen cluster labels were identified in the English literature, predominantly encompassing drugs (#0 smilax glabra, #1 rhizome smilacis glabrae, #6 matrie injection, # 7astilbin, # 10engeletin, etc.). Mechanism research (# 2bone resorption, # 3network pharmacology, # 4 molecular docking, # 5nf kappa b, #16 serum); Disease research (# 9 gout); Method study (#11 pretreatment and drying method).

**Figure 8:**
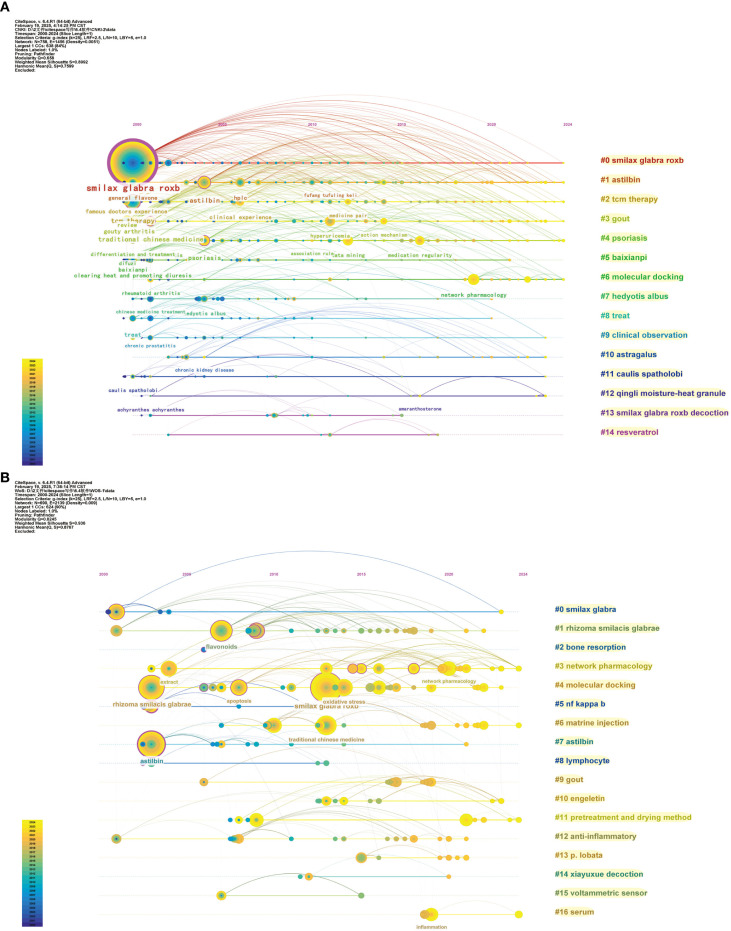
Timeline of key words in Chinese **(A)** and English **(B)** literature on SGR.

Chronological keyword clustering analysis can be employed to investigate relationships and developmental processes among research hotspots in this domain (Jiahe et al., 2025). **Figure 8A**, **8B** depicts the chronology of keyword clustering. From 2000 to 2024, the clustering tags #0, #1, #2, #3, #4, and #6 in Chinese literature signify that fundamental research on the pharmacodynamic substances of TCM SGR and its application in treating gout and psoriasis has been a prominent research focus. Conversely, the English literature predominantly emphasizes the advancement and refinement of SGR analysis methodologies and pharmacological mechanisms of SGR.

#### 3.5.4 Keyword emergence analysis

**Figure 9:**
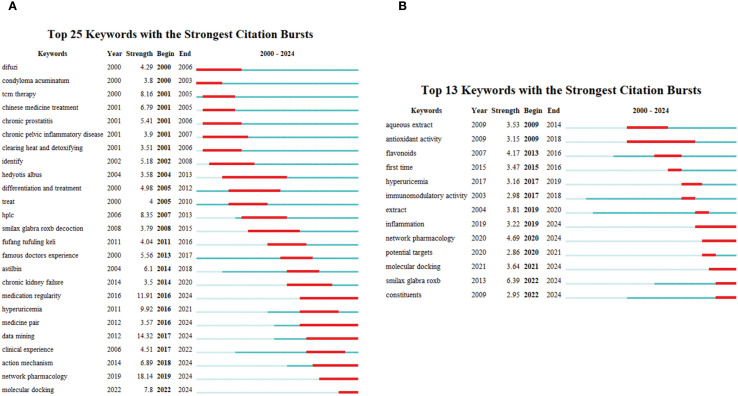
Keyword emergence patterns in Chinese **(A)** and English **(B)** related literature on SGR.

Keyword emergence analysis is a technique employed to examine the changes in frequency of keywords over a designated timeframe, which can reveal research hotspots, trends, and evolving frontiers within a scholarly domain (L. Yang et al., 2023). This study performed a keyword emergence analysis of the pertinent literature, and the results are illustrated in **Figure 9**. **Figure 9A** shows the keyword emergence map of Chinese literature, comprising 25 emergent terms. The three keywords exhibiting the highest emergence intensity were network pharmacology (18.14), data mining (14.32), and medication regularity (11.91). Since 2016, terms such as drug use rule, drug pair, data mining, mechanism of action, and molecular docking of SGR have surfaced, signifying that research on drug pairs and the mechanism of SGR has attracted considerable attention. **Figure 9B** illustrates the keyword emergence map of SGR-related English literature, comprising nine emergent terms. The three keywords exhibiting the highest breakout strength were Smilax glabra (6.39), network pharmacology (4.69), and flavonoids (4.17). The keywords inflammation, network pharmacology, and SGR constituents have emerged as prominent research trends since 2019. This signifies that the principal research trajectory emphasizes animal experimentation integrated with molecular bioinformatics to investigate the pharmacological mechanisms of SGR.

## 4 Discussion

In recent years, interest in the study of Chinese medicine, particularly SGR and several other Chinese medicinal formulations, has intensified. This emerging trend is evidenced in the increasing quantity of publications issued annually. This study performed a bibliometric analysis of all English and Chinese publications pertaining to SGR from 2000 to 2024, utilizing CiteSpace software. We employed bibliometric techniques to visualize and analyze publication volume, author co-citation, publishing organizations, and keywords. English publications on SGR-related studies had a consistent increasing tendency, whilst Chinese journals remained comparatively constant. China is a significant contributor to research, hosting numerous prominent research teams. Moreover, the extensively referenced papers illustrate the present research status and developmental patterns, serving as a crucial reference for analyzing the development prospects of SGR.

An analysis of published papers indicates that the quantity of SGR-related articles in Chinese literature hovers around 60 per year, exhibiting a generally consistent trend. Conversely, English literature demonstrates a distinct upward trajectory, indicating that international academics are progressively concentrating on SGR. Overall, the volume of published papers in Chinese literature significantly exceeds that in English literature, indicating that research on SGR is more active in China, whereas relevant studies worldwide remain limited, although there is a noteworthy upward trend. In summary, SGR has gained great scientific interest and attracted significant attention both domestically and globally.

According to Price’s Law (Benjun et al., 2024), the formula for calculating the minimum number of publications necessary for a core author is $N=0.749\times \sqrt{n_{max}}$ , where n_max_ is the number of publications by the most prolific author. An author is deemed a core contributor in the field if their publication count surpasses N. In this study, the team led by Sun Weifeng resulted in the publication of numerous papers primarily centered on the extraction process of compound SGR granules (Wei et al., 2013, 2014; Zhigang et al., 2011) and the treatment of hyperuricemia and gout (Jing et al., 2023, 2019, 2021; Tian and Weifeng, 2012; Xiaoyi et al., 2016). The analysis revealed nmax=21 and N≈3.43, indicating that authors with four or more publications constituted the core group, comprising a total of 32 individuals. Zhang and QingFeng’s team led English literature in published papers, focusing on the quality evaluation of SGR and the analysis of its active constituents (Zhang and Cheung, 2011; Zhang et al., 2013a, 2013b, 2009a, 2009b), nmax=10, N≈2.37. The authors who had published three or more publications in this field were considered core authors, totaling 11 individuals.

In the analysis of research institutions in the Chinese literature, the Beijing University of Chinese Medicine was the most distinguished with 26 articles published. Subsequently, the Guangzhou University of Chinese Medicine published 25 publications, Shandong University of Chinese Medicine published 23 articles, and Zhejiang University of Chinese Medicine published 22 articles. The most prominent publishing institution in English literature was Zhejiang Chinese Medical University, with 16 articles, followed by Guangzhou University of Chinese Medicine with 12 articles, Jiangxi Agricultural University with 11 articles, and Nanjing University of Chinese Medicine with 7 articles. This data indicate that Chinese institutions significantly contribute to the overall development of the field and play a crucial role. The accompanying map depicts the tight collaboration across diverse institutions, particularly in their affiliations with regional Chinese medicine universities and associated hospitals. Collaborative cooperation and resource allocation among these entities are crucial for fostering further progress in this domain.

The analysis of institutions and authors shows that Chinese research institutions outperform other countries, a phenomenon that can be attributed to two main reasons: First, SGR has a long history of use as a medicinal plant in China. A review of the journals that have published SGR-related research papers over the past 25 years shows that the majority of these journals have been published mainly in journals of traditional Chinese medicine, journals of practical TCM, and journals of ethnopharmacology, and that China, as a pioneer in the field of SGR research, has carried out a variety of long-term studies on the theories of the properties of traditional Chinese medicines, the rules of medication, and pharmacological studies (Guo et al., 2024; Hua et al., 2018), so that the Chinese language literature dominates the number of papers. Secondly, with the deepening of research on it in other countries, there has been a gradual increase in the isolation and characterization of new chemical components of SGR and the study of new pharmacological mechanisms of action such as antihypertension (Gegentana et al., 2024; Yang et al., 2024). Therefore, a comprehensive analysis of the development trend of SGR in China and internationally is an important reference value for the research and development of new dosage forms of its future drugs, innovative medicines, and health foods.

Keyword co-occurrence analysis provides an intuitive overview of principal themes and frequently occurring keywords within a specific research field. The prevalence of these keywords indicates the primary research emphasis and focal points within the field. This investigation identified the prevalent terms in Chinese literature as SGR (677 times), TCM therapy (147 times), and gout (87 times). The predominant terms in English literature were SGR. (59 times), astilbin (40 times), and rhizoma smilacis glabrae (38 times). This paper examines the investigation of SGR and its active components, along with the therapy of gout utilizing SGR and its pharmacological mechanisms.

The global emerging keyword co-occurrence map illustrates that Initial and intermediate career research on SGR concentrated on identifying its constituents and conducting pharmacodynamic studies (Chen et al., 2014; Zhang et al., 2013), whereas subsequent investigations emphasize pharmacological activity and mechanisms of action, employing network pharmacology and other bioinformatics technologies alongside experimental methodologies (Guo et al., 2024; Zhao et al., 2025). This progression illustrates the advancement of research in this domain, evolving from preliminary component analysis to an emphasis on mechanisms, which corresponds with contemporary trends in Chinese medicine research. Interdisciplinary methodologies, including network pharmacology, bioinformatics, and molecular biology, have been thoroughly utilized to investigate the mechanisms of action of the active ingredients in TCM. Furthermore, the examination of these active constituents involves intricate systems that engage various targets and pathways, requiring assistance from contemporary science and technology. Therefore, future research should persist in fostering multidisciplinary collaboration to advance the comprehensive exploration of the active constituents of TCM. In summary, although the current research covers a variety of fields such as drug research, pharmacodynamic research, and pharmacological research, SGR still suffers from the disadvantages of poor oral bioavailability, easy structural transformation of active ingredients, and difficulty in obtaining the active ingredients with high purity. Therefore, future research on SGR should be conducted more from the perspectives of enhancing the rationality of clinical use and improving the bioavailability of the drug to address the current focused problems that have not yet been overcome.

## 5 Strengths and limitations

Based on objective quantitative analysis, bibliometric research can proficiently assess the developmental patterns and focal points within the SGR domain. This provides valuable insights for scholars seeking new avenues; yet, certain restrictions must be acknowledged. First, our literature review was confined to English publications retrieved from the WoSCC database, perhaps resulting in an insufficient assemblage of pertinent studies. Second, owing to the dynamic nature of database updates, we exclusively incorporated literature published from 2000 to 2024. As a result, prior studies may have been omitted, while recently released, high-quality literature may have been overlooked.

## 6 Conclusion

This study employed citespace software to do a thorough review of Chinese and English literature on SGR from 2000 to 2024. The annual number of publications, co-authors, collaborating institutions, keyword co-occurrence, clustering, and emerging trends were illustrated through graphs and tables to investigate the research directions, hotspots, and developmental trends in this domain. The results indicate that the investigation of pharmaceuticals, their active ingredients, disease treatment, and pharmacological mechanisms alongside bioinformatics continues to be a prominent research focal point. In recent years, as research on SGR has deepened, transdisciplinary methodologies have emerged as a dominant trend. A thorough examination of TCM components has clarified their mechanisms of action, which is highly significant for future research on SGR. This study offers a thorough analysis of global SGR research, identifying potential limitations and emerging research directions, thereby facilitating the exploration of SGR’s future development through detailed insights and practical research guidelines.
